# The Role of Arterioles and the Microcirculation in the Development of Vasospasm after Aneurysmal SAH

**DOI:** 10.1155/2014/253746

**Published:** 2014-05-11

**Authors:** Masato Naraoka, Naoya Matsuda, Norihito Shimamura, Kenichiro Asano, Hiroki Ohkuma

**Affiliations:** Department of Neurosurgery, Hirosaki University, 5-Zaihuchou, Hirosaki, Aomori Prefecture 036-8562, Japan

## Abstract

Cerebral vasospasm of the major cerebral arteries, which is characterized by angiographic narrowing of those vessels, had been recognized as a main contributor to delayed cerebral ischemia (DCI) in subarachnoid hemorrhage (SAH) patients. However, the CONSCIOUS-1 trial revealed that clazosentan could not improve mortality or clinical outcome in spite of successful reduction of relative risk in angiographic vasospasm. This result indicates that the pathophysiology underlying DCI is multifactorial and that other pathophysiological factors, which are independent of angiographic vasospasm, can contribute to the outcome. Recent studies have focused on microcirculatory disturbance, such as microthrombosis and arteriolar constriction, as a factor affecting cerebral ischemia after SAH. Reports detecting microthrombosis and arteriolar constriction will be reviewed, and the role of the microcirculation on cerebral ischemia during vasospasm after SAH will be discussed.

## 1. Introduction

Cerebral vasospasm after aneurysmal SAH was demonstrated for the first time by Ecker and Riemenschneider in 1951 [1]. They indicated that arterial narrowing of the major arteries near the circle of Willis could be seen on cerebral angiography in six of 29 cases of SAH. Since then, cerebral vasospasm of the major cerebral arteries has been recognized as a main contributor to delayed neurological deterioration of SAH patients, and this deterioration is referred to as delayed ischemic neurological deficits (DIND) or delayed cerebral ischemia (DCI). A large number of investigations have been carried out in an attempt to clarify the mechanism of sustained constriction of arterial smooth muscle cells and to develop treatment methods to ameliorate it. In contrast, there has been little attention paid to cerebral microcirculation as a factor affecting DCI.

However, the CONSCIOUS-1 trial (use of clazosentan to overcome neurological ischemia and infarction occurring after SAH), which was a randomized, blinded clinical trial using an endothelin antagonist, clazosentan, revealed that clazosentan could not improve mortality or clinical outcome in spite of a successful reduction of relative risk of angiographic vasospasm by 65% [2]. This result indicates that the pathophysiology underlying DCI is multifactorial and that other pathophysiological factors, which are independent of angiographic vasospasm, can contribute to the outcome [3]. Recently, the focus has shifted to cerebral microcirculatory disturbance, a pathophysiological factor other than vasospasm of the major cerebral arteries that was not considered important in the past. The trend toward studying the microcirculation during cerebral vasospasm, as it emerged in the past and presently, is reviewed and the significance of microcirculatory disturbance is discussed.

## 2. Narrowing of the Large Cerebral Arteries and Cerebral Ischemia

The first step in establishing the concept of cerebral vasospasm was based on the recognizing that the narrowing of the major cerebral arteries correlates well with cerebral ischemia. As an early study, Fletcher et al. found that angiographic vasospasm relates to poor neurologic status and focal neurologic deficits. Angiographic vasospasm was described as segmental or diffused and found to be present three weeks after SAH in 39 of 100 patients [4]. Fisher et al. graded angiographic vasospasm from grades 0 to IV according to the diameter of the residual lumen of the proximal segments of the anterior and middle cerebral arteries. Of 31 patients with grade III or grade IV, 80% of cases developed DIND. Of 19 patients with a lesser grade, none developed DIND [3]. Saito et al. reported that angiographic vasospasm was sometimes correlated with neurological signs and symptoms, while cases with no neurological deterioration exhibited only slight angiographic vasospasm. They classified angiographic vasospasm as extensive diffuse, multisegmental, or local and indicated that the mortality rates associated with these types were 45, 19, and 10%, respectively [5]. Weir et al. measured eight arterial points on 627 angiograms from 293 patients and indicated that the patients with the most angiographic vasospasm had significantly higher mortality rates than those with the least angiographic vasospasm [6].

Thus, most studies before 1980 had pointed to an association of the degree of angiographic vasospasm with neurological impairments or patient outcome. In addition, a correlation was indicated between angiographic vasospasm and cerebral blood flow (CBF) detected by SPECT and emission CT with 133Xe inhalation [7, 8]. These findings suggested that luminal decrease of the major cerebral arteries is the main factor causing reduced CBF and ischemic symptoms.

## 3. Previous Concepts regarding DCI Pathogenesis

Novel pathological mechanisms have been suggested, including damage to cerebral tissue in the first 72 hours after aneurysm rupture (early brain injury), cortical spreading depression (CSD), microcirculatory dysfunction, and microthrombosis [9–12].

### 3.1. Early Brain Injury

Early brain injury is a term that refers to the damage done to the brain in the first 72 hours after the initial bleeding [13]. The release of arterial blood into the subarachnoid space is accompanied by intense headache and an acute increase in intracranial pressure, often causing intracranial circulatory arrest and loss of consciousness [14, 15]. The mechanisms of the resulting early brain injury are dominated by cell death, blood-brain barrier (BBB) disruption, and brain edema [16]. Animal models show BBB disruption as early as 30 minutes after cortical SAH [17], and the leakage of large molecules remains high within the first 48 hours of bleeding [18]. It seems probable that the physiological changes occurring at the onset of DCI directly influence the severity of later ischemic complications in patients after SAH. Although experimental results done mainly in rats seem to mirror measurements in patients with aneurysmal rebleeding and intracranial pressure (ICP) monitoring in situ, the majority of animal models rely on iatrogenic damage to cerebral vessels to simulate aneurysm rupture and induce subarachnoid bleeding [19].

### 3.2. Cortical Spreading Depression (CSD) and DCI

The first CSD was demonstrated experimentally in the 1940s in rabbit cortex. Originally, the process was regarded as an experimental artifact that had little relevance to neurological disease in humans. Recently, however, there has been a resurgence of interest in CSD. In the last few years, CSD has been identified as a potential pathophysiological mechanism contributing to DCI. The term describes a depolarization wave in cerebral grey matter that propagates across the brain at 2–5 mm/min and results in depression of evoked and spontaneous EEG activity [20]. It has been implicated in the pathophysiology of a number of neurological diseases, including malignant hemispheric stroke [21], traumatic brain injury [22], and DCI after SAH [23]. In SAH, there is good evidence from animal models and patient studies that CSDs occur after the initial bleed [23, 24]. Spreading ischemia was first described in a rat model of DCI and it results from local microvascular dysfunction. It is thought that, with each depolarization, there is an associated, profound hypoperfusion of the cortex due to vasoconstriction [25].

The incidence of CSDs measured in patients after SAH seems to correlate with the time frame for the development of DCI, with data from one study demonstrating that 75% of all CSDs recorded occurred between the fifth and seventh days after SAH [26]. CSDs also seem to occur in the absence of angiographic vasoconstriction. Despite placement of nicardipine pellets around the middle cerebral artery to minimize proximal vasoconstriction, spontaneous depolarizations still occurred in 10 of 13 patients [27], casting further doubt on the exact nature of the contribution of proximal vessel constriction to DCI.

### 3.3. Microcirculatory Dysfunction and Microthrombosis

Clinical investigations had suggested that intraparenchymal small vessels are dilated after SAH in order to compensate for reduced peripheral perfusion pressure caused by vasospasm of the major arteries. Grubb et al. investigated CBF, CMRO2, and cerebral blood volume (CBV) by using positron emission tomography (PET) in SAH patients. A decrease in CBF and CMRO2 and an increase in CBV were seen in patients with poor-grade SAH and severe symptomatic vasospasm. The poor grade patients with symptomatic vasospasm showed reduced CBF under 20 mL/100 g/min and increased CBV over 2 mL/100 g/min. They suggested that cerebral vasospasm of large arteries is accompanied by a massive dilation of the intraparenchymal vessels [28]. A CBF study using single photon emission computed tomography showed that CBF did not increase by administering acetazolamide in SAH patients [29]. Furthermore, a transcranial Doppler sonography (TCD) study revealed that hypercapnia does not decrease flow velocities in the middle cerebral arteries or the internal carotid arteries of patients with cerebral vasospasm [30]. These decreased reactivities to vasodilating stimuli were thought to be due to a lack of response by small vessels because of their maximal dilation in an attempt to maintain sufficient CBF in the face of severe vasospasm [29, 30]. Studies using several imaging techniques in patients with SAH [31–34] and in animal models of SAH [35–37] have suggested the existence of microvessel constriction and microthrombi formation after SAH. In clinical studies, large artery angiographic vasospasm on admission angiography is also an adverse prognostic factor for outcome [38, 39]. One limitation of these imaging studies is that they examined microvessels visible on the pial surface. Activation of the coagulation cascade, impairment of the fibrinolytic cascade, activation of inflammation, and endothelium related processes may all play a role [40]. Histological studies on a prechiasmatic injection SAH model showed that microthromboemboli are abundant in brain parenchyma [41–43]. Sehba et al. demonstrated the involvement of platelet aggregation and neutrophil infiltration of the observed microvascular injury in the vascular perforation model [44, 45].

In the main, the concept that small vessels dilate during cerebral vasospasm was considered to be valid, and several reports that showed microthrombosis or narrowing of small vessels, as described later, were not considered significant.

Postmortem studies of SAH patients have demonstrated evidence of microthrombi. Patients with DCI have significantly more microthrombi in areas showing cerebral infarction than those patients who die from aneurysmal rebleed or hydrocephalus [46]. Microthrombosis also correlated with the amount of overlying free subarachnoid blood and clinical and pathological signs of ischemia [47]. Interestingly, a postmortem study into microthrombosis after SAH showed that, while cortical ischemic lesions were present in 77% of patients, there was no significant association between the presence of these lesions and angiographic vasospasm or aneurysm location [48].

## 4. Reassessment of the Role of the Microcirculation in Cerebral Ischemia during Vasospasm

During the period when the concepts described above prevailed, several reports indicated a discrepancy between the degree of angiographic vasospasm and DCI or decreased CBF. There had been several reports showing that clinical symptoms of DCI or decreased CBF can occur without angiographic evidence of vasospasm [8, 49, 50] and that severe angiographic vasospasm is often found in the patients without obvious DCI or decreased CBF [51–53]. However, these concepts were not considered important before the 2000s.

Recently, reports showing that angiographic vasospasm is not always correlated with DCI, cerebral infarction, or CBF have been accumulating. Rabinstein et al. indicated that the location of cerebral infarction in SAH patients cannot be predicted in one-quarter to one-third of patients by angiogram or TCD [54]. Weidauer et al. revealed that cortical band-like infarction develops without evidence of severe angiographic vasospasm in SAH patients [55, 56]. And some clinical studies indicated that angiographic vasospasm of large arteries on admission is an adverse prognostic factor for outcome [38, 39].

Dissociation between angiographic vasospasm and outcome after SAH was noted in the CONSCIOUS-1 trial, which became epoch making in terms of changing the concept of vasospasm [2]. After the CONSCIOUS-1 trial, reports of a discrepancy between angiographic vasospasm and CT hypodensities or regional hypoperfusion have been increasing [57, 58]. One of the explanations for these observations could be that the cerebral microcirculation and its regulatory mechanisms are directly affected by SAH and cause DCI. For microcirculatory dysfunction during vasospasm after SAH, mainly microthrombosis and microarterial constriction have been investigated [12, 59].

## 5. Microthrombosis

### 5.1. Detection of Microthrombi

Adhesion of aggregated platelets or mural thrombi at the site of vasospasm of major cerebral arteries had been indicated in early reports [60–62]. Reports on the action of these platelets and thrombi suggest that arteries are narrowed by mural thrombi as well as by proliferative organic changes in the arterial wall and such narrowing is often confused with prolonged vasospasm [62, 63]. Also, aggregated platelets may release vasoactive substances that produce smooth muscle constriction that results in arterial narrowing [60].

Microthrombi were detected for the first time in 1983 by Suzuki et al. in a patient who died due to cerebral vasospasm after SAH. Light microscopy of sectioned slices showed that the parenchymal microthrombi consisted mostly of white thrombi composed of aggregated platelets, with fibrin also observed in some of these. They suggested that microthrombi could be contributors to cerebral ischemia during vasospasm [64]. Later, they confirmed the role of microthrombi in cerebral vasospasm by investigating six patients who died after SAH. Of the six patients, four died of DCI and two of rebleeding or acute hydrocephalus. Compared to the latter two patients, the other four patients showed significantly more microthrombi of intraparenchymal small vessels in clinically ischemic regions and in areas showing cerebral infarction on CT scan. They concluded that the significant regional correlation of thrombi distribution and DCI suggests a close relationship between them [46]. This is supported by a recent autopsy study investigating 29 SAH patients by Stein et al. They revealed a strong correlation between microclot burden and DCI, as patients with clinical or radiological evidence of DCI had, in the mean, significantly more microclot burdens than patients without DCI. And there was also a significant association between microclot burden and histological evidence of ischemia [47].

Furthermore, microthrombi have also been shown in experimental studies. Seven days after SAH, microthrombi in the cerebral and cerebellar cortex can be found in the rat SAH model, in which SAH is produced by blood injection into the prechiasmatic cistern [65]. Peak microthrombi formation is seen 48 hours after SAH in an endovascular perforation SAH model of mouse [66]. Microthrombi in the parenchymal arterioles are also seen 48 hours after SAH in a prechiasmatic blood injection model in mice [12]. In vivo fluorescence microscopy using a mouse endovascular perforation SAH model revealed that 30% of pial arterioles were occluded by microthrombi, which demonstrates that microthrombosis is not a histological artifact but also occurs after SAH in vivo [37]. Accumulation of this clinical and experimental evidence seems to strongly affirm the concept that microthrombi comprise one important factor affecting cerebral ischemia after SAH.

### 5.2. Pathophysiology and Mechanism of Microthrombus Formation (Figure 1)

Endothelial function of major cerebral arteries is known to be disturbed during vasospasm after SAH [67]. Prostaglandin I2 is synthesized in the endothelial cells and inhibits the circulating platelets from adhering and aggregating to endothelial cells, and its synthesis at the major cerebral arteries is disturbed during vasospasm [68].

Ohkuma et al. revealed that antiplatelet-aggregating activity in the endothelial cells of the basilar artery is impaired in feline two-hemorrhage SAH models. After production of SAH, adenosine diphosphate was infused into the basilar artery via the right vertebral artery to activate circulating platelets,and many platelets were observed adhering or aggregating on the luminal surface four to seven days after SAH. They suggested that this impairment may be involved in inducing cerebral ischemia during cerebral vasospasm by causing platelet adhesion and aggregation [69]. They also indicated increased platelet function in the case of DCI. Sequential changes of platelet aggregability and beta-thromboglobulin and thromboxane B2 concentrations in blood samples from the internal jugular and peripheral vein were investigated, and platelet function in patients with symptomatic vasospasm showed more enhancements in blood from the internal jugular vein than in blood from a peripheral vein. These results suggest that platelets are activated through vasospastic major arteries and that the resulting increased tendency for thrombus formation may affect the patient's prognosis during the advanced stage [70].

Some reports suggest that a functional disturbance of microvessels itself can be a causative factor for microthrombi. Sabri et al. indicated that decreased NO and increased P-selectin in the endothelium of arterioles is a mechanism for microthrombosis [12]. Direct observation of pial arterioles after endovascular perforation in an SAH model in mice indicated that arteriolar constriction is followed by local formation of microthrombi, and the frequency of arteriolar microthrombosis correlates with the degree of its constriction [37]. This finding also suggests that functional damage in arterioles can cause local thrombus formation.

Changes in the blood coagulation cascade can also contribute to microthrombi formation [40]. Several studies show that the coagulation cascade is already activated within a few days after SAH before the occurrence of vasospasm. This early activation of the coagulation pathway is an early predictor of the occurrence of DCI and infarction after SAH [40]. Levels of platelet activating factor in internal jugular venous blood start to increase within four days after SAH [71]. Increased levels of Von Willebrand factor within 72 hours after SAH correlate with the occurrence of DCI and poor outcome after SAH [72]. These factors are considered to induce platelet activation in the early stage of SAH [40]. Furthermore, in the acute phase after SAH, concentrations of tissue factor, which is the primary initiator of coagulation through activating thrombin, are elevated in the cerebrospinal fluid (CSF) [73].

Increased levels of fibrinopeptide A, an alternative marker of thrombin generation, within two days after SAH, are also associated with cerebral infarction after SAH [74]. And patients with DCI after SAH have significantly higher levels of plasminogen activator inhibitor-1 antigen in the CSF as compared with patients without DCI, suggesting that overactive inhibition of fibrinolysis is associated with DCI [74]. Fibrin formation then also takes part in increased coagulation activity. Therefore, a systemic coagulation cascade activated in the early stage, endothelial dysfunction of microvessels, and platelet activation through vasospastic large arteries are, together, involved in microthrombi formation and its effect DCI [75].

### 5.3. Prevention of Microthrombosis

Trapidil, an antagonist and selective synthesis inhibitor of thromboxane A_2_, was administrated in a series of 20 cases of SAH. Vasospasm was demonstrated by angiography in nine of these cases, but only two of the nine showed mild signs of cerebral ischemia, which suggests the significance in symptomatic vasospasm of thrombus formation by platelet aggregation and the effectiveness of trapidil as a preventive agent [76]. They also tried OKY-046, an imidazole derivative and a thromboxane syntheses inhibitor; it was studied cooperatively at ten neurosurgical services [77] or at 48 neurosurgical services in Japan in double-blind fashion [78]. Both trials showed the usefulness of OKY-046 for the prevention of symptomatic vasospasm and support the hypothesis that cerebral microthrombosis plays an important role in the pathogenesis of cerebral vasospasm.

Aspirin was used in two trials; both studies, however, included a small number of patients and failed to show its efficacy for the prevention of symptomatic vasospasm or improved outcome [79, 80]. Dipyridamole also failed to show efficacy for reducing the incidence of ischemic deficits [81]. However, a systematic review including five studies indicates that antiplatelet drugs reduce the risk of DCI in patients with SAH [82]. Recent experimental studies showed simvastatin and mutant thrombin-activated urokinase-type plasminogen activator are effective in reducing microthrombi [65, 66]. Recent clinical studies have also indicated that cilostazol, which is a selective inhibitor of phosphodiesterase 3, an antiplatelet agent marketed in Japan, and which is used to treat ischemic symptoms of peripheral vascular disease, can decrease the incidence of symptomatic vasospasm, severe angiographic vasospasm, vasospasm-related new cerebral infarctions, and poor outcome in patients with aneurysmal SAH [83–85].

## 6. Vasoconstriction of Arterioles

### 6.1. Detection of Arteriolar Constriction

Microvessel constriction after SAH was demonstrated by Herz et al. in a vascular micropuncture model of SAH in guinea pigs. Their experiments also suggested that a chemomechanical mechanism might be involved in the vasoconstriction of pial microvessels [35]. However, the observation period of microvessel constriction was limited to the unlra-early stage after SAH or stimulation. Hart performed morphometric determinations of the external diameter and wall thickness of intraparenchymal arterioles two hours after blood was injected into the cisterna magna of cats and observed a decreased external arteriolar diameter accompanied by an increased wall thickness. He suggested these changes were caused by constriction of arterioles [86]. However, serial morphological changes in the intraparenchymal arterioles days after SAH coincident with delayed cerebral vasospasm of large cerebral arteries remain unclear in those investigations.

Morphological changes of parenchymal arterioles during the vasospasm period after SAH were examined for the first time by Ohkuma et al. In a canine double hemorrhage SAH model, corrosion casts of arterioles showed tapered narrowing with folding after SAH. Morphometric examination by light microscopy showed a significant decrease in the internal diameter of arterioles associated with a significant increase in wall thickness three and seven days after SAH. These results suggest that constriction of intraparenchymal arterioles occurs after SAH and may contribute to delayed cerebral ischemia [87]. They also revealed that the same changes in perforating arteries occurred in basilar arteries by using the same technique and the same animal model [88].

After that, pial microvessels were directly observed under an operative microscope during aneurysm surgery. Uhl et al. examined pial microcirculation in humans using orthogonal polarization spectral imaging. Patients with SAH who were operated on within three days after SAH showed that capillary density was significantly decreased and small arteries and arterioles of the cortical surface exhibited vasospasm. They suggested those changes may contribute to the initial clinical symptoms and may have an influence on the clinical postoperative course [32]. By using the same technology, Pennings et al. tested contractile responses of the cerebral arterioles in 16 patients who underwent aneurysm surgery. Ten patients were operated on early (within 48 hours after bleeding) and six underwent late surgery. The contractile response of the arterioles to hyperventilation was increased, accompanied by a bead-string constriction pattern in patients operated on early compared to those in late surgeries and in controls [33]. They also revealed the microvascular responses to papaverine in patients undergoing aneurysm surgery. In patients with SAH, unpredictable response patterns to papaverine were observed with “rebound” vasoconstriction. They considered that the results suggest increased contractility of the microcirculation [34].

In addition, recent experimental studies showed the same results. Friedrich et al. examined pial arterioles three, six, and 72 hours after SAH by using in vivo fluorescence microscopy in an endovascular perforation SAH model of mice and found that arterioles constricted by 22% to 33% up to three days after SAH, which demonstrates that SAH induces microarterial constrictions and microthrombosis in vivo [37]. They suggested that these findings may explain the early cerebral perfusion pressure-independent decrease in CBF after SAH and therefore microarterial constrictions and microthrombosis may serve as novel targets for the treatment of early perfusion deficits after SAH.

### 6.2. Pathophysiology and Mechanism of Microvessel Constriction (Figure 1)

Arterioles can constrict in response to various vasoactive substances [89]. Spasmogenic substances derived from subarachnoid clots can then easily affect pial arterioles. And they also affect intraparenchymal arterioles by penetrating into the perivascular space [87]. Another possible factor is vasoactive substances that act on the wall of arterioles from inside the vessels. Circulating platelets activated by endothelial dysfunction through the vasospastic large cerebral arteries liberate vasospastic substances, such as thromboxane A_2_, serotonin, or adenosine diphosphate, which can induce smooth muscle constriction in peripheral arterioles [70]. Endothelial dysfunction of arterioles, such as decreased NO production, can cause arteriolar constriction [12].

Arteriolar constriction is now believed to play an important role in DCI after SAH, but there are several problems to be solved. Microvessel constriction during the period of vasospasm has not been fully proved in humans. Ohkuma et al. measured cerebral circulation time (CCT) and CBF in 24 cases of aneurysmal SAH. CCT was divided into proximal CCT, which was the circulation time through the extraparenchymal large arteries, and peripheral CCT, which was the circulation time through the intraparenchymal small vessels. Peripheral CCT showed a strong inverse correlation with rCBF. Even in nonmild or moderate angiographical vasospasm, prolonged peripheral CCT was clearly associated with decreased rCBF [90]. In other words, rCBF decreased in spite of the absence of angiographical vasospasm in the major artery. These results suggested that microvessel constriction prolonged peripheral CCT and decreased rCBF independently. It was also considered that the cause of peripheral CCT prolongation was based not only on microvessel constriction but also on a microthrombosis.

As far as other problems to be solved, we still need clarification on how many vessels are affected, which microvessels are mostly affected, and how much microvessel constriction is associated with cerebral ischemia. Those problems should be addressed in the future in order to improve outcome for SAH patients by establishing prevention and treatment of arteriolar constriction.

## Figures and Tables

**Figure 1:**
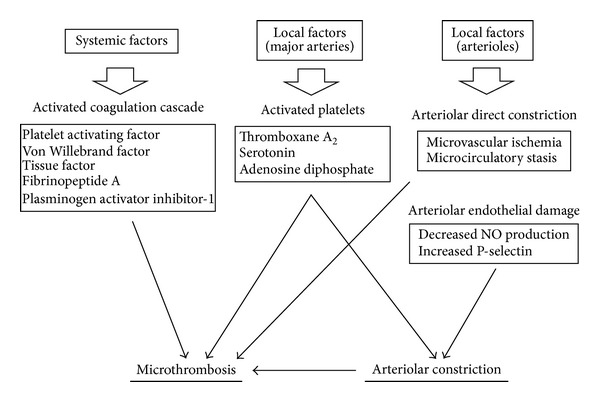
Pathophysiology and mechanism of microthrombus formation and arteriolar constriction.

## References

[1] Ecker A, Riemenschneider PA (1951). Arteriographic demonstration of spasm of the intracranial arteries, with special reference to saccular arterial aneurysms. *Journal of neurosurgery*.

[2] Macdonald RL, Kassell NF, Mayer S (2008). Clazosentan to overcome neurological ischemia and infarction occurring after subarachnoid hemorrhage (CONSCIOUS-1): randomized, double-blind, placebo-controlled phase 2 dose-finding trial. *Stroke*.

[3] Fisher CM, Roberson GH, Ojemann RG (1977). Cerebral vasospasm with ruptured saccular aneurysm–the clinical manifestations. *Neurosurgery*.

[4] Fletcher TM, Taveras JM, Pool JL (1959). Cerebral vasospasm in angiography for intracranial aneurysms. Incidence and significance in one hundred consecutive angiograms. *Archives of Neurology*.

[5] Saito I, Ueda Y, Sano K (1977). Significance of vasospasm in the treatment of ruptured intracranial aneurysms. *Journal of Neurosurgery*.

[6] Weir B, Grace M, Hansen J, Rothberg C (1978). Time course of vasospasm in man. *Journal of Neurosurgery*.

[7] Mickey B, Vorstrup S, Voldby B (1984). Serial measurement of regional cerebral blood flow in patients with SAH using 133Xe inhalation and emission computerized tomography. *Journal of Neurosurgery*.

[8] Jakobsen M, Overgaard J, Marcussen E, Enevoldsen EM (1990). Relation between angiographic cerebral vasospasm and regional CBF in patients with SAH. *Acta Neurologica Scandinavica*.

[9] Macdonald RL, Pluta RM, Zhang JH (2007). Cerebral vasospasm after subarachnoid hemorrhage: the emerging revolution. *Nature Clinical Practice Neurology*.

[10] Rowland MJ, Hadjipavlou G, Kelly M, Westbrook J, Pattinson KTS (2012). Delayed cerebral ischaemia after subarachnoid haemorrhage: looking beyond vasospasm. *British Journal of Anaesthesia*.

[11] Zhang JH, Pluta RM, Hansen-Schwartz J (2009). Cerebral vasospasm following subarachnoid hemorrhage: time for a new world of thought. *Neurological Research*.

[12] Sabri M, Ai J, Lakovic K, D'abbondanza J, Ilodigwe D, Macdonald RL (2012). Mechanisms of microthrombi formation after experimental subarachnoid hemorrhage. *Neuroscience*.

[13] Kusaka G, Ishikawa M, Nanda A, Granger DN, Zhang JH (2004). Signaling pathways for early brain injury after subarachnoid hemorrhage. *Journal of Cerebral Blood Flow and Metabolism*.

[14] Grote E, Hassler W (1988). The critical first minutes after subarachnoid hemorrhage. *Neurosurgery*.

[15] Voldby B, Enevoldsen EM (1982). Intracranial pressure changes following aneurysm rupture. Part I. Clinical and angiographic correlations. *Journal of Neurosurgery*.

[16] Cahill WJ, Calvert JH, Zhang JH (2006). Mechanisms of early brain injury after subarachnoid hemorrhage. *Journal of Cerebral Blood Flow and Metabolism*.

[17] Doczi T (1985). The pathogenetic and prognostic significance of blood-brain barrier damage at the acute stage of aneurysmal subarachnoid haemorrhage. Clinical and experimental studies. *Acta Neurochirurgica*.

[18] Germanò A, D’Avella D, Imperatore C, Caruso G, Tomasello F (2000). Time-course of blood-brain barrier permeability changes after experimental subarachnoid haemorrhage. *Acta Neurochirurgica*.

[19] Fujii M, Yan J, Rolland WB, Soejima Y, Caner B, Zhang JH (2013). Early brain injury, an evolving frontier in subarachnoid hemorrhage research. *Translational Stroke Research*.

[20] Leao AAP (1947). Further observations on the spreading depression of activity in the cerebral cortex. *Journal of Neurophysiology*.

[21] Dohmen C, Sakowitz OW, Fabricius M (2008). Spreading depolarizations occur in human ischemic stroke with high incidence. *Annals of Neurology*.

[22] Strong AJ, Fabricius M, Boutelle MG (2002). Spreading and synchronous depressions of cortical activity in acutely injured human brain. *Stroke*.

[23] Dreier JP, Major S, Manning A (2009). Cortical spreading ischaemia is a novel process involved in ischaemic damage in patients with aneurysmal subarachnoid haemorrhage. *Brain*.

[24] Dreier JP, Woitzik J, Fabricius M (2006). Delayed ischaemic neurological deficits after subarachnoid haemorrhage are associated with clusters of spreading depolarizations. *Brain*.

[25] Shin HK, Dunn AK, Jones PB, Boas DA, Moskowitz MA, Ayata C (2006). Vasoconstrictive neurovascular coupling during focal ischemic depolarizations. *Journal of Cerebral Blood Flow and Metabolism*.

[26] Bosche B, Graf R, Ernestus R-I (2010). Recurrent spreading depolarizations after subarachnoid hemorrhage decreases oxygen availability in human cerebral cortex. *Annals of Neurology*.

[27] Woitzik J, Dreier JP, Hecht N (2012). Delayed cerebral ischemia and spreading depolarization in absence of angiographic vasospasm after subarachnoid hemorrhage. *Journal of Cerebral Blood Flow and Metabolism*.

[28] Grubb RL, Raichle ME, Eichling JO, Gado MH (1977). Effects of subarachnoid hemorrhage on cerebral blood volume, blood flow and oxygen utilization in humans. *Journal of Neurosurgery*.

[29] Shinoda J, Kimura T, Funakoshi T, Araki Y, Imao Y (1991). Acetazolamide reactivity on cerebral blood flow in patients with subarachnoic haemorrhage. *Acta Neurochirurgica*.

[30] Hassler W, Chioffi F (1989). CO_2_ reactivity of cerebral vasospasm after aneurysmal subarachnoid haemorrhage. *Acta Neurochirurgica*.

[31] Romano JG, Forteza AM, Concha M (2002). Detection of microemboli by transcranial Doppler ultrasonography in aneurysmal subarachnoid hemorrhage. *Neurosurgery*.

[32] Uhl E, Lehmberg J, Steiger HJ (2003). Intraoperative detection of early microvasospasm in patients with subarachnoid hemorrhage by using orthogonal polarization spectral imaging. *Neurosurgery*.

[33] Pennings FA, Bouma GJ, Ince C (2004). Direct observation of the human cerebral microcirculation during aneurysm surgery reveals increased arteriolar contractility. *Stroke*.

[34] Pennings FA, Albrecht KW, Muizelaar JP, Schuurman PR, Bouma GJ (2009). Abnormal responses of the human cerebral microcirculation to papaverin during aneurysm surgery. *Stroke*.

[35] Herz DA, Baez S, Shulman K (1975). Pial microcirculation in subarachnoid hemorrhage. *Stroke*.

[36] Sun BL, Zheng CB, Yang MF, Yuan H, Zhang SM, Wang LX (2009). Dynamic alterations of cerebral pial microcirculation during experimental subarachnoid hemorrhage. *Cellular and Molecular Neurobiology*.

[37] Friedrich B, Müller F, Feiler S, Schöller K, Plesnila N (2012). Experimental subarachnoid hemorrhage causes early and long-lasting microarterial constriction and microthrombosis: an in-vivo microscopy study. *Journal of Cerebral Blood Flow and Metabolism*.

[38] Baldwin ME, Macdonald RL, Huo D (2004). Early vasospasm on admission angiography in patients with aneurysmal subarachnoid hemorrhage is a predictor for in-hospital complications and poor outcome. *Stroke*.

[39] Salary M, Quigley MR, Wilberger JE (2007). Relation among aneurysm size, amount of subarachnoid blood, and clinical outcome. *Journal of Neurosurgery*.

[40] Vergouwen MDI, Vermeulen M, Coert BA, Stroes ESG, Roos YBWEM (2008). Microthrombosis after aneurysmal subarachnoid hemorrhage: an additional explanation for delayed cerebral ischemia. *Journal of Cerebral Blood Flow and Metabolism*.

[41] Sabri M, Ai J, Knight B (2011). Uncoupling of endothelial nitric oxide synthase after experimental subarachnoid hemorrhage. *Journal of Cerebral Blood Flow and Metabolism*.

[42] Sabri M, Ai J, MacDonald RL (2011). Dissociation of vasospasm and secondary effects of experimental subarachnoid hemorrhage by clazosentan. *Stroke*.

[43] Sabri M, Ai J, Marsden PA, Macdonald RL (2011). Simvastatin re-couples dysfunctional endothelial nitric oxide synthase in experimental subarachnoid hemorrhage. *PLoS ONE*.

[44] Sehba FA, Mostafa G, Friedrich V, Bederson JB (2005). Acute microvascular platelet aggregation after subarachnoid hemorrhage. *Journal of Neurosurgery*.

[45] Friedrich V, Flores R, Muller A, Bi W, Peerschke EIB, Sehba FA (2011). Reduction of neutrophil activity decreases early microvascular injury after subarachnoid haemorrhage. *Journal of Neuroinflammation*.

[46] Suzuki S, Kimura M, Souma M, Ohkima H, Iwabuchi T, Shimiz u T (1990). Cerebral microthrombosis in symptomatic cerebral vasospasm—a quantitative histological study in autopsy cases. *Neurologia Medico-Chirurgica*.

[47] Stein SC, Browne KD, Chen XH, Smith DH, Graham DI (2006). Thromboembolism and delayed cerebral ischemia after subarachnoid hemorrhage: an autopsy study. *Neurosurgery*.

[48] Neil-Dwyer G, Lang DA, Doshi B, Gerber CJ, Smith PWF (1994). Delayed cerebral ischaemia: the pathological substrate. *Acta Neurochirurgica*.

[49] Graham DI, Macpherson P, Pitts LH (1983). Correlation between angiographc vasospasm, hematoma, and ischemic brain damage following SAH. *Journal of Neurosurgery*.

[50] Kawamura S, Sayama I, Yasui N, Uemura K (1992). Sequential changes in cerebral blood flow and metabolism in patients with subarachnoid haemorrhage. *Acta Neurochirurgica*.

[51] Proust F, Debono B, Gérardin E (2002). Angiographic cerebral vasospasm and delayed ischemic deficit on anterior part of the circle of Willis: usefulness of transcranial Doppler. *Neurochirurgie*.

[52] Geraud G, Tremoulet M, Guell A, Bes A (1984). The prognostic value of noninvasive CBF measurement in subarachnoid hemorrhage. *Stroke*.

[53] Powsner RA, O’Tuama LA, Jabre A, Melhem ER (1998). SPECT imaging in cerebral vasospasm following subarachnoid hemorrhage. *Journal of Nuclear Medicine*.

[54] Rabinstein AA, Friedman JA, Weigand SD (2004). Predictors of cerebral infarction in aneurysmal subarachnoid hemorrhage. *Stroke*.

[55] Weidauer S, Lanfermann H, Raabe A, Zanella F, Seifert V, Beck J (2007). Impairment of cerebral perfusion and infarct patterns attributable to vasospasm after aneurysmal subarachnoid hemorrhage: a prospective MRI and DSA Study. *Stroke*.

[56] Weidauer S, Vatter H, Beck J (2008). Focal laminar cortical infarcts following aneurysmal subarachnoid haemorrhage. *Neuroradiology*.

[57] Dhar R, Scalfani MT, Blackburn S, Zazulia AR, Videen T, Diringer M (2012). Relationship between angiographic vasospasm and regional hypoperfusion in aneurysmal subarachnoid hemorrhage. *Stroke*.

[58] Ibrahim GM, Weidauer S, Vatter H, Raabe A, MacDonald RL (2012). Attributing hypodensities on CT to angiographic vasospasm is not sensitive and unreliable. *Stroke*.

[59] Østergaard L, Aamand R, Karabegovic S (2013). The role of the microcirculation in delayed cerebral ischemia and chronic degenerative changes after subarachnoid hemorrhage. *Journal of Cerebral Blood Flow & Metabolism*.

[60] Alksne JF, Branson PJ (1980). Pathogenesis of cerebral vasospasm. *Neurological Research*.

[61] Fein JM, Flor WJ, Cohan SL, Parkhurst J (1974). Sequential changes of vascular ultrastructure in experimental cerebral vasospasm. Myonecrosis of subarachnoid arteries. *Journal of Neurosurgery*.

[62] Mizukami M, Kin H, Araki G (1976). Is angiographic spasm real spasm?. *Acta Neurochirurgica*.

[63] Someda K, Morita K, Kawamura Y, Matsumura H (1979). Intimal change following subarachnoid hemorrhage resulting in prolonged arterial luminal narrowing. *Neurologia Medico-Chirurgica*.

[64] Suzuki S, Suzuki M, Iwabuchi T, Kamata Y (1983). Role of multiple cerebral microthrombosis in symptomatic cerebral vasospasm: with a case report. *Neurosurgery*.

[65] Wang Z, Chen G, Zhu WW, Bian JY, Shen XO, Zhou D (2010). Influence of simvastatin on microthrombosis in the brain after subarachnoid hemorrhage in rats: a preliminary study. *Annals of Clinical and Laboratory Science*.

[66] Pisapia JM, Xu X, Kelly J (2012). Microthrombosis after experimental subarachnoid hemorrhage: time course and effect of red blood cell-bound thrombin-activated pro-urokinase and clazosentan. *Experimental Neurology*.

[67] Sasaki T, Kassell NF (1990). The role of endothelium in cerebral vasospasm. *Neurosurgery clinics of North America*.

[68] Tokoro K (1984). Cerebral vasospasm and lipoperoxide damage. Morphological localization and measurement of lipoperoxide in prolonged cerebral vasospasm. *Neurological Surgery*.

[69] Ohkuma H, Ogane K, Fujita S, Manabe H, Suzuki S, Rosenblum WI (1993). Impairment of anti-platelet-aggregating activity of endothelial cells after experimental subarachnoid hemorrhage. *Stroke*.

[70] Ohkuma H, Suzuki S, Kimura M, Sobata E (1991). Role of platelet function in symptomatic cerebral vasospasm following aneurysmal subarachnoid hemorrhage. *Stroke*.

[71] Hirashima Y, Nakamura S, Endo S, Kuwayama N, Naruse Y, Takaku A (1997). Elevation of platelet activating factor, inflammatory cytokines, and coagulation factors in the internal jugular vein of patients with subarachnoid hemorrhage. *Neurochem. Res*.

[72] Frijns CJM, Kasius KM, Algra A, Fijnheer R, Rinkel GJE (2006). Endothelial cell activation markers and delayed cerebral ischaemia in patients with subarachnoid haemorrhage. *Journal of Neurology, Neurosurgery and Psychiatry*.

[73] Hirashima Y, Nakamura S, Suzuki M (1997). Cerebrospinal fluid tissue factor and thrombin-antithrombin III complex as indicators of tissue injury after subarachnoid hemorrhage. *Stroke*.

[74] Kasuya H, Shimizu T, Takakura K (1998). Thrombin activity in CSF after SAH is correlated with the degree of SAH, the persistence of subarachnoid clot and the development of vasospasm. *Acta Neurochirurgica*.

[75] Ikeda K, Asakura H, Futami K, Yamashita J (1997). Coagulative and fibrinolytic activation in cerebrospinal fluid and plasma after subarachnoid hemorrhage. *Neurosurgery*.

[76] Suzuki S, Sobata E, Iwabuchi T (1981). Prevention of cerebral ischemic symptoms in cerebral vasospasm with trapidil, an antagonist and selective synthesis inhibitor of thromboxane A2. *Neurosurgery*.

[77] Suzuki S, Iwabuchi T, Tanaka T (1985). Prevention of cerebral vasospasm with OKY-046 an imidazole derivative and a thromboxane synthetase inhibitor. A preliminary co-operative clinical study. *Acta Neurochirurgica*.

[78] Suzuki S, Sano K, Handa H (1989). Clinical study of OKY-046, a thromboxane synthetase inhibitor, in prevention of cerebral vasospasms and delayed cerebral ischaemic symptoms after subarachnoid haemorrhage due to aneurysmal rupture: a randomized double-blind study. *Neurological Research*.

[79] Mendelow AD, Stockdill G, Steers AJW (1982). Double-blind trial of patients receiving tranexamic acid for subarachnoid haemorrhage. *Acta Neurochirurgica*.

[80] Hop JW, Rinkel GJE, Algra A, Berkelbach Van Der Sprenkel JW, van Gijn J (2000). Randomized pilot trial of postoperative aspirin in subarachnoid hemorrhage. *Neurology*.

[81] Shaw MD, Foy PM, Conway M (1985). Dipyridamole and postoperative ischemic deficits in aneurysmal subarachnoid hemorrhage. *Journal of Neurosurgery*.

[82] Mees SMD, Rinkel GJE, Hop JW, Algra A, van Gijn J (2003). Antiplatelet therapy in aneurysmal subarachnoid hemorrhage a systematic review. *Stroke*.

[83] Suzuki S, Sayama T, Nakamura T (2011). Cilostazol improves outcome after subarachnoid hemorrhage: a preliminary report. *Cerebrovascular Diseases*.

[84] Senbokuya N, Kinouchi H, Kanemaru K (2013). Effects of cilostazol on cerebral vasospasm after aneurysmal subarachnoid hemorrhage: a multicenter prospective, randomized, open-label blinded end point trial. *Journal of Neurosurgery*.

[85] Niu PP, Yang G, Xing YQ, Guo ZN, Yang Y (2014). Effect of cilostazol in patients with aneurysmal subarachnoid hemorrhage: a systematic review and meta-analysis. *Journal of the Neurological Sciences*.

[86] Hart MN (1980). Morphometry of brain parenchymal vessels following subarachnoid hemorrhage. *Stroke*.

[87] Ohkuma H, Itoh K, Shibata S, Suzuki S (1997). Morphological changes of intraparenchymal arterioles after experimental subarachnoid hemorrhage in dogs. *Neurosurgery*.

[88] Ohkuma H, Suzuki S (1999). Histological dissociation between intra- and extraparenchymal portion of perforating small arteries after experimental subarachnoid hemorrhage in dogs. *Acta Neuropathologica*.

[89] Golding EM, S. Robertson C, Bryan RM (1998). Comparison of the myogenic response in rat cerebral arteries of different calibers. *Brain Research*.

[90] Ohkuma H, Manabe H, Tanaka M, Suzuki S (2000). Impact of cerebral microcirculatory changes on cerebral blood flow during cerebral vasospasm after aneurysmal subarachnoid hemorrhage. *Stroke*.

